# In-vitro assessment of appropriate hydrophilic scaffolds by co-electrospinning of poly(1,4 cyclohexane isosorbide terephthalate)/polyvinyl alcohol

**DOI:** 10.1038/s41598-020-76471-x

**Published:** 2020-11-12

**Authors:** Abdul Salam, Muhammad Qamar Khan, Tufail Hassan, Nafees Hassan, Ahsan Nazir, Tanveer Hussain, Musaddaq Azeem, Ick Soo Kim

**Affiliations:** 1 Nanotechnology Research Group, Department of Textile and Clothing, Faculty of Engineering and Technology, National Textile University Karachi Campus, Industrial Area Korangi, Karachi, 74900 Pakistan; 2 Department of Textile Chemical Processing, Faculty of Engineering and Technology, National Textile University, Faisalabad, Pakistan; 3 Department of Material Engineering, Faculty of Textile Engineering, Technical University of Liberec, studentska’ 1402/2, 46117 Librerec 1, Czech Republic; 4 Division of Frontier Fiber, Institute of Fiber Engineering, Interdisciplinary Cluster for Cutting Edge Research (ICCER), Faculty of Textile Sciences, Shinshu University, Tokida 3-15-1, Ueda, Nagano 386-8567 Japan

**Keywords:** Biotechnology, Materials science

## Abstract

Textile-based Scaffolds preparation has the attractive features to fulfill the stated and implied needs of the consumer but there are still challenges of stability, elongation, appreciable bio-compatibility, and stated hydrophilic behavior. To overcome these challenges, the authors tried to fabricate a scaffold by blending of two highly biocompatible polymers; polyvinyl alcohol and poly(1,4 cyclohexane isosorbide terephthalate) through co-electrospinning. The resultant scaffold by the stated innovative approach evaluated from different characterizations such as dimensional stability/morphology was evaluated by scanning electron microscopy, chemical interactions by that Fourier transmission infrared spectra, wetting behavior was analyzed by a static angle with a contact angle meter from drop method, elongation was examined by tensile strength tester and in-vitro assessment was done by MTT analysis. Based on verified results, it was concluded that PVA/PICT scaffold has a potential for dual nature of hydrophilicity & hydrophobicity and appreciable cell culture growth, stated dimensional stability and suitable elongation as per requirements of the nature of scaffold.

## Introduction

Nanofibers are those fibers that have a diameter in the nanometric range that is, less than 100 nm. These nanofibers possessed different properties as compared to the microfibers. When the diameter of the polymeric fiber’s changes from micrometric scale to the nanometric scale, there is a significant change in the properties of the fibers such as surface to volume ratio increased, surface functional properties changes, mechanical properties such as stiffness, the strength of the fibers changed. These properties make nanofibers superior from the other fibers. Due to these superior properties nanofibers are extensively used in different fields for different application purposes, such as medical applications (wound dressing^1,2^ drug delivery system^3–5^), aerospace applications^6,7^, electronics applications (transistors, capacitors, energy storage devices)^8,9^. Different techniques have been used to produce nanofibers. The drawing technique is only used for the viscoelastic polymeric material, usually, the tip of the micropipette dipped in the polymeric solution and drawn mechanically from the solution results in the formation of nanofibers^10^. In template synthesis, nano-porous tubular templates (molds) are used to produce solid or hollow nanofibers. Usually, polymeric materials drew through these molds by some mechanical action result in the formation of nanofibers. The advantage of this technique is that it can be used to produce nanofibers from any material like metals, conductive polymers, and semiconductor polymers. The drawback of this technique is that continuous nanofibers cannot be produced^11,12^. Another simple but time taken technique is the preparation of nanofibers by using the phase separation method. Usually, a gel of the polymer solution is formed by storing the solution at gelation temperature, this gel is socked into the distilled water for exchange of solvent, removal of water results in the formation of nanofibers matrix. This method is beneficial at the lab scale^13^. In the self-assembly technique, pre-existing components arranged themselves into a specific pattern. As similar to the phase separation technique, the self-assembly technique is a time-consuming process.

Techniques described previously for the preparation of the nanofibers have their advantages and disadvantages, but these techniques cannot be used for the bulk production of the nanofibers. However, electrospinning is only the technique that can be used for the continuous bulk production of nanofibers. Electrospinning is a versatile and widely used technique to produce nanofibers in the nanometric range by using an electric field generated at different voltages. Usually, the electrospinning apparatus contains a high voltage power supply, a spinneret of specific diameter through which polymer solution extruded and a collecting system on which produced nanofibers are collected. However, a single type of polymer can be used to produces nanofibers from electrospinning. To produce mixed fibers produced from different polymers, different approaches are used. Co-electrospinning is a sophisticated approach to produce the mixed composite fibers from two different polymers. In this technique, one electrospinning is combined with other electrospinning. Designated needles are used for each polymer while the position of the needle is opposite to each other. This approach is easy to produce the composite nanofibers and fibers are connected either by covalent bonding or via entanglements^14^. Another approach for producing mixed fibers is by combining the electrospinning with melt-blown methods. Usually, the microfibers produced from the melt-blown combine with the electrospinning to produce the composite material^15^. Generally, mixed fibers are used to obtain dual properties from any material.

**Figure 1:**
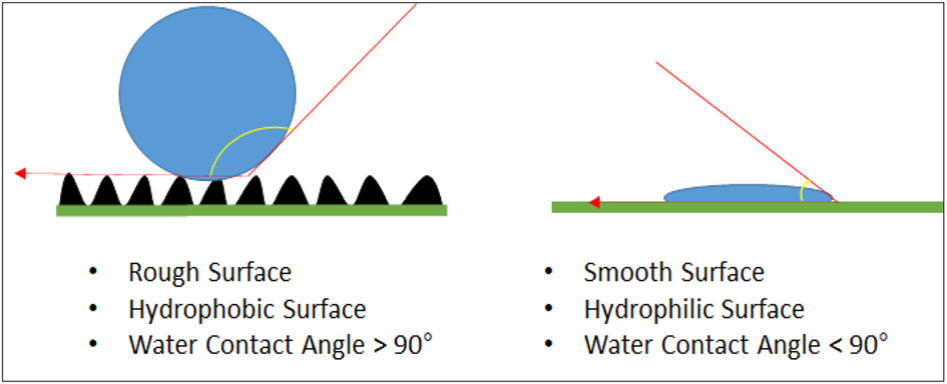
Surface mechanism of the hydrophobic and Hydrophilic behavior.

Hydrophobicity and hydrophilicity is another property of different polymers. Due to this property such, polymers can be used for different applications. If the contact angle between surface and droplet is greater than 90°, then such a surface is known as the hydrophobic surface. For a super-hydrophobic surface, the contact angle should be greater than 150°, in such a case water droplet attains spherical shape and roll off the surface^16–18^. The contact angle can be increased with the roughness of the surface and air trapped between the water and the surface. In a super-hydrophobic surface, the grip between the surface and the dirt particle is smaller than the grip between the particle and droplet; as a result, the particle is captured by the droplet of water, results in the cleaning of the surface as mentioned in the Fig. 1. In case of hydrophilic surface, the contact angle between the surface and the water droplet is less than 90°, as a result when the water droplet falls in the surface of material it will be adsorbed on the surface of material instead of rolling off. Different researchers perform their study for the development of hydrophobic and super-hydrophobic and hydrophilic surfaces. Electrospinning of lot of hydrophobic drugs can be done with organic solvent^19^.

Similarly, electrospinning of lots of hydrophilic drugs can be done with liquid phase PVA or PEO. Liu et al. found that polyimide nanofibers having a diameter in the range of 300 nm to 400 nm show excellent hydrophobic properties at with contact angle of 140°. They have found that these nanofibers can be used for self-cleaning surfaces, self-cleaning solar panel surfaces^20^. Kang et al. determined that the contact angle in the case of polystyrene was 138.1° and 138.8° when it was electrospun in tetrahydrofuran and chloroform respectively. When such polystyrene was electrospun in N,N-dimethylformamide solvent, this angle was considerably changed to 154.2°^21^. Zheng et al. reported that the fibers electrospun with co-polymer poly(styrene styrene-b-dimethylsiloxane) show excellent superhydrophobicity with the contact angle of 160° when the diameter of the fibers was in the range of 150 nm to 40 nm^22^.

Apart from the above discussion, this study is focused on the development of nano-composite by using co-electrospinning of PVA and PICT polymers. The resultant nano-composite material will have both hydrophobic and hydrophilic properties because PVA polymer is hydrophilic in nature and PICT is hydrophobic in nature so when these two polymers electrospun together the nanofibers will possess dual properties. Such a nano-composite can be used for the scaffold and filtration.

## Experimental

### Materials

Polyvinyl alcohol (PVA) (Mw: 85,000–124,000, 89% hydrolyzed was purchased from Sigma-Aldrich, USA. Poly (1, 4-cyclohexanedimethylene isosorbide terephthalate) (PICT) was kindly supplied by SK chemicals, the Republic of Korea as pellet type. Trifluoroacetic acid (99.9%) and chloroform (99%) were purchased from Wako Pure Chemical Industries, Ltd, Japan, and deionized water was used.

### Fabrication of scaffold

**Figure 2:**
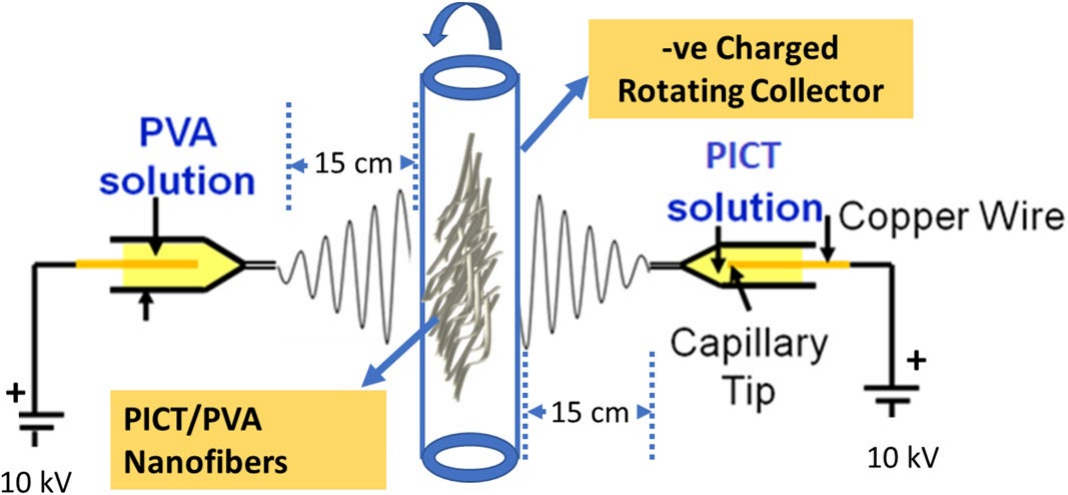
Illustration scheme of fabrication of scaffold.

To prepare the spinning solution, PICT 10% wt was dissolved in trifluoroacetic acid and chloroform in ratios of 1:3 and PVA solution was prepared in which PVA powder 10% wt was dissolved in deionized water and stirred at 70 °C for 6 h. The scaffold was prepared by co-electrospinning as mentioned in Fig. 2. The solutions were loaded in the two different plastic syringes connected with capillary tips having an inner diameter of 0.60 mm, in which an electrode of Cu wire was adjusted in each plastic syringes. The distance from capillary tips to the collector was 15 cm and the supply of voltage was 10 kV and a flow rate of 0.6 ml h^−1^.

### Characterizations

#### Morphology analysis

The surface morphology of the scaffold was analyzed by SEM (JSM-5300, JEOL Ltd., Japan) accelerated with a voltage of 12 kV. The average diameter of nanofibers was determined from 200 measurements of the random nanofibers using image analysis software (Image J, version 1.49)^24^.

#### Chemical interactions analysis

The chemical interactions between PICT and PVA nanofibers were studied by Fourier transform infrared (FT-IR) spectra achieved by IR Prestige-21 (Shimadzu, Japan). The spectra were recorded from 400–4000 cm^−1^ with a resolution of 4 cm^−1^ and the addition of 128 scans^23,24^.

#### Tensile strength universal testing machine

The tensile strength test was performed to check the mechanical behavior of nanofibers by using the Universal Testing Machine (Tesilon RTC 250A, A&D Company Ltd., Japan), the 5 specimens for each sample were prepared under a cross-head speed of 1 mm/min at room temperature and the values of stress and strain were calculated by the following formulas (1) and (2) respectively^24^.

1 $$\text{Stress }\left(\text{MPa}\right)=\frac{\text{All \,values\, of\, load}\left(\text{N}\right)}{\text{Area }((\text{width }\times \text{ thickness})^2)}$$

2 $$\text{Strain }\left(\text{\%}\right)=\frac{\text{change\, in\, length}(\Delta\text{ l})}{\text{initial \,length}(\text{l})} \times 100$$

#### Water contact angle meter

To investigate the wetting behavior of PICT, PVA and PICT/PVA nanofibers, a static angle with a contact angle meter by drop method (Kyowa Interface Science, Japan), was used^25^. As per ASTM D7334, a web having 2.54 cm × 5.08 cm dimensions from each sample was prepared and fixed at a glass strap where a drop of water from the needle of the water contact angle meter was dropped at the fixed web on five different places and behavior was examined. Images were automatically captured by the water contact angle meter because a camera is part of this machine.

#### In-vitro assessment

To investigate the cell’s attachment behavior of resultant scaffolds, an MTT study was done for seven days in line L929 with a two-time repeat in triplicate for each sample and cell line. MTT {3-(4,5-dimethylthiazol-2-yl)-2,5-diphenyltetrazolium bromide} is a dye compound that is used as an indicator for assessing the growth of the cells. Due to the high light sensitivity of MTT reagent, the MTT assay is done in the dark. The darker solution corresponds to a higher number of cell growth. After sterilization, the samples were cultured for up to 7 days in DMEM/F12 with 10% FBS for L929. The cell viability was measured in the percentage of the negative control. After each day, 15% of medium culture containing *Thiazolyl Blue Tetrazolium Bromide* (MTT; 5 mg/ml) was added to each well and incubated in 37 °C with 5% CO_2_ for 12 h. After this, the medium was removed, and samples were washed with PBS. Formosan crystals were dissolved in 400 µl DMSO. The amount of 200 µl of each solution was transferred into the new 96 wells and the absorbance was measured at 570 nm. To measure the dye absorption by the scaffolds, all samples without any cells were immersed in a medium culture with the addition of MTT, then washed with DMSO and absorbance was read at 570 nm. For the estimation of the number of cells adhering to the scaffolds, the absorbance of scaffolds was subtracted from the absorbance in the presence of cell line L929^26^.

## Results and discussions

### Morphology analysis

**Figure 3:**
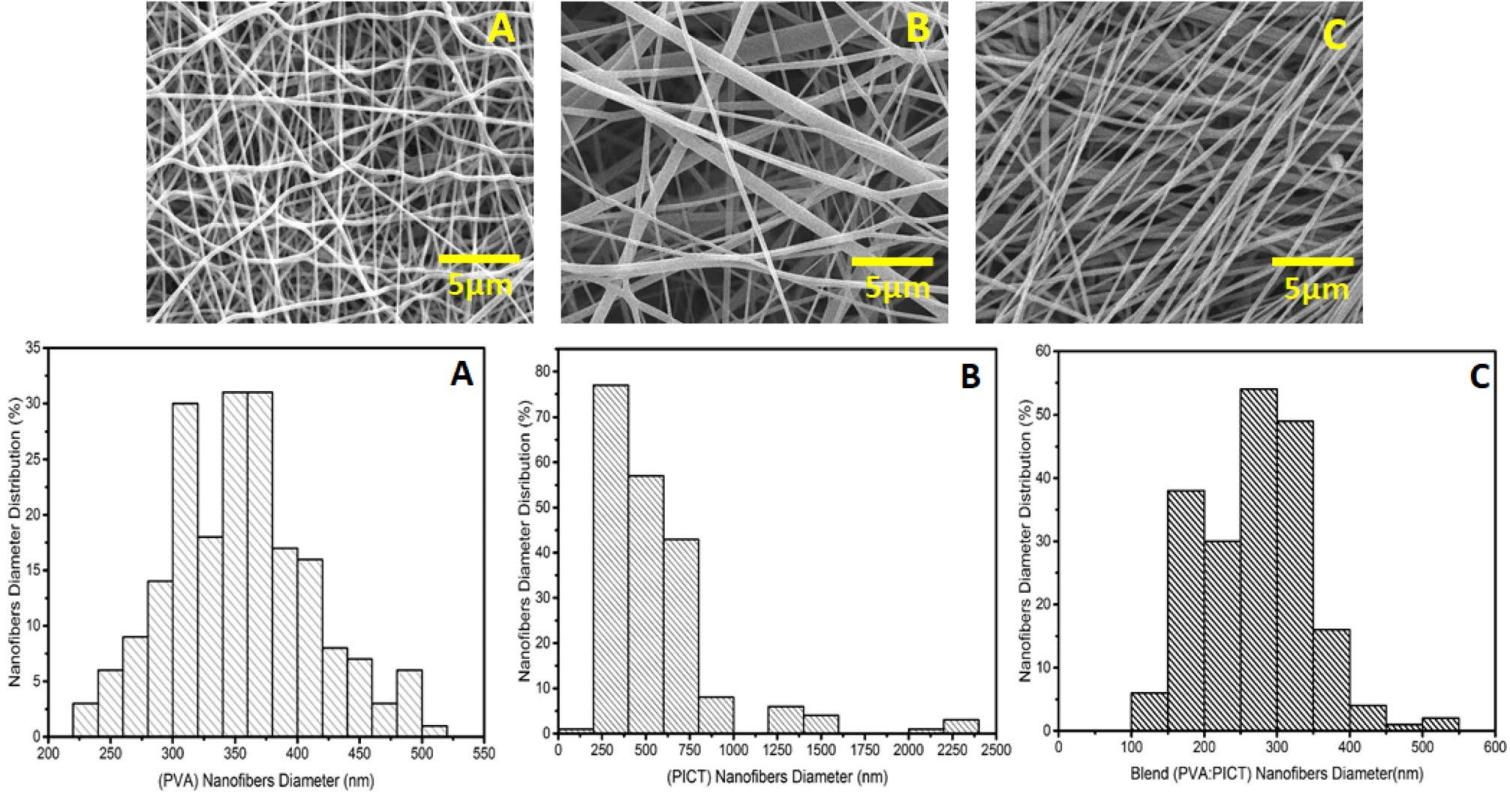
SEM images and diameter distributions of (**A**) PVA electrospun nanofibers, (**B**) PICT electrospun nanofibers and (**C**) Blend (PVA: PICT) electrospun nanofibers.

The Surface morphology of all the electrospun nanofibers was analyzed by using a scanning electron microscope (SEM) as shown in Fig. 3. In all cases (PVA, PICT and Blend), it can be observed that very smooth electrospun nanofibers formed without any formation of beads. The diameter of all the resultant electrospun nanofibers was analyzed by using Image J software. Figure 3A shows the diameter distributions of PVA electrospun nanofibers. The major axis (X-axis) represents the diameter of nanofibers while the minor axis (Y-axis) contains the diameter distribution of electrospun nanofibers. In the case of PVA electrospun nanofibers, the average diameter lies in the range of 353 nm and having a standard deviation value in the range of 58 nm. Figure 3B contains the histogram of PICT electrospun nanofibers it can be seen in the histogram that the average diameter of electrospun nanofibers lies in the range of 560 nm with the standard deviation value of 350 nm. Figure 3C shows the histogram of blend (PVA: PICT) electrospun nanofibers, the average diameter of the electrospun nanofibers lies in the range of 268 nm with the standard deviation of 74 nm. From all the histograms it can be concluded that the PICT electrospun nanofibers with the highest values of standard deviation show more variation in the structure, while the PVA electrospun nanofibers have an excellent surface structure with a fine diameter as compare to PICT and blend (PVA: PICT) electrospun nanofibers but the blended form is better than neat PICT nanofibers.

### EDS spectra analysis

**Figure 4:**
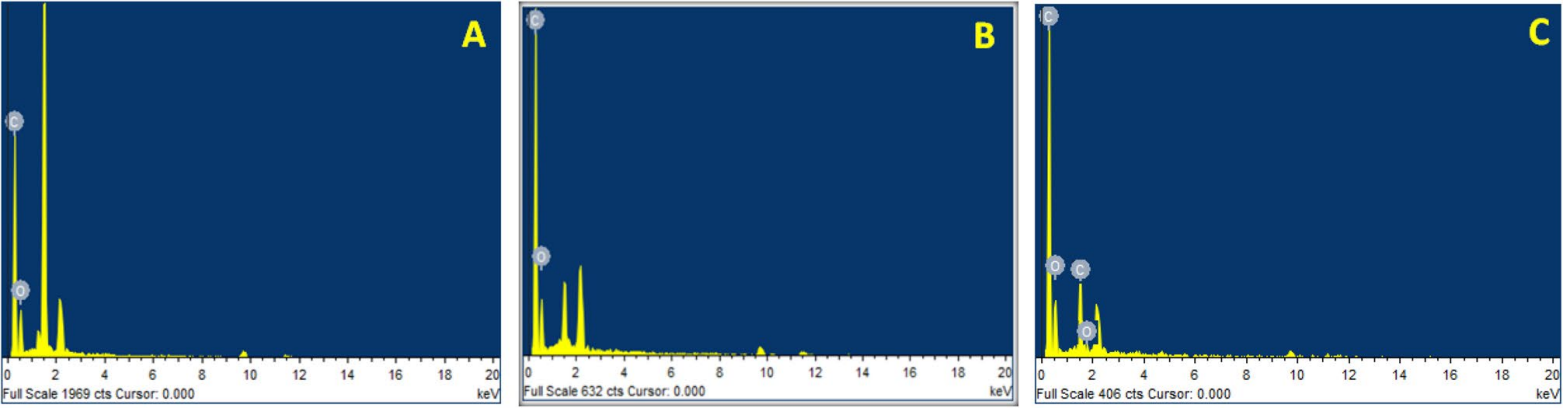
EDS spectra of (**A**) neat PVA, (**B**) neat PICT and (**C**) blended (PVA: PICT) nanofibers.

Elemental analysis of pure PVA, pure PICT, and Blend (PVA: PICT) electrospun nanofibers was carried out with the help of energy-dispersive X-ray spectroscopy (EDS). In Fig. 4 elemental spectra of pure PVA, pure PICT and blend nanofibers are shown. Elemental spectra of pure PVA, pure PICT showed only the peaks of C and O. There is no other peak of any foreign element present in pure PVA and pure PICT spectra. Blend electrospun nanofibers also show the peaks of C and O but in this case, the intensity of C and O peaks increased due to the mixing of PVA and PICT nanofibers. No doubt that Fig. 4A,B, have similar peaks but the number of peaks varies in the blended sample that is Fig. 4C. In Fig. 4C we can see that the detected number of peaks for O is two while in Fig. 4A,B there is one peak of O in each case. Similarly, the number of detected C peaks also increases in the case of a blend (Fig. 4C) while in the case of Fig. 4A,B only one peak of Carbon detected. The possible reason behind this could be that by mixing two polymers the concentration of carbon and Oxygen in the blend material increase. The highest peak appears (in Fig. 4A) at 1.5 keV indicates the carbon element, similar peak also appears in the spectrum C but in this case, the peak height of the Carbon element decreases. Because the EDS spectrum normally displays peaks corresponding to the energy levels or which the most X-rays had been received. Moreover, the carbon atom has three different isotopes C12, C13 and C14, and each isotope has different absorption and excitation properties, this could be the possibility that the peak height may be decreased due to the formation of different isotopes.

The results confirm that there is no interference of any impurity in the prepared samples.

### Chemical interactions analysis

**Figure 5:**
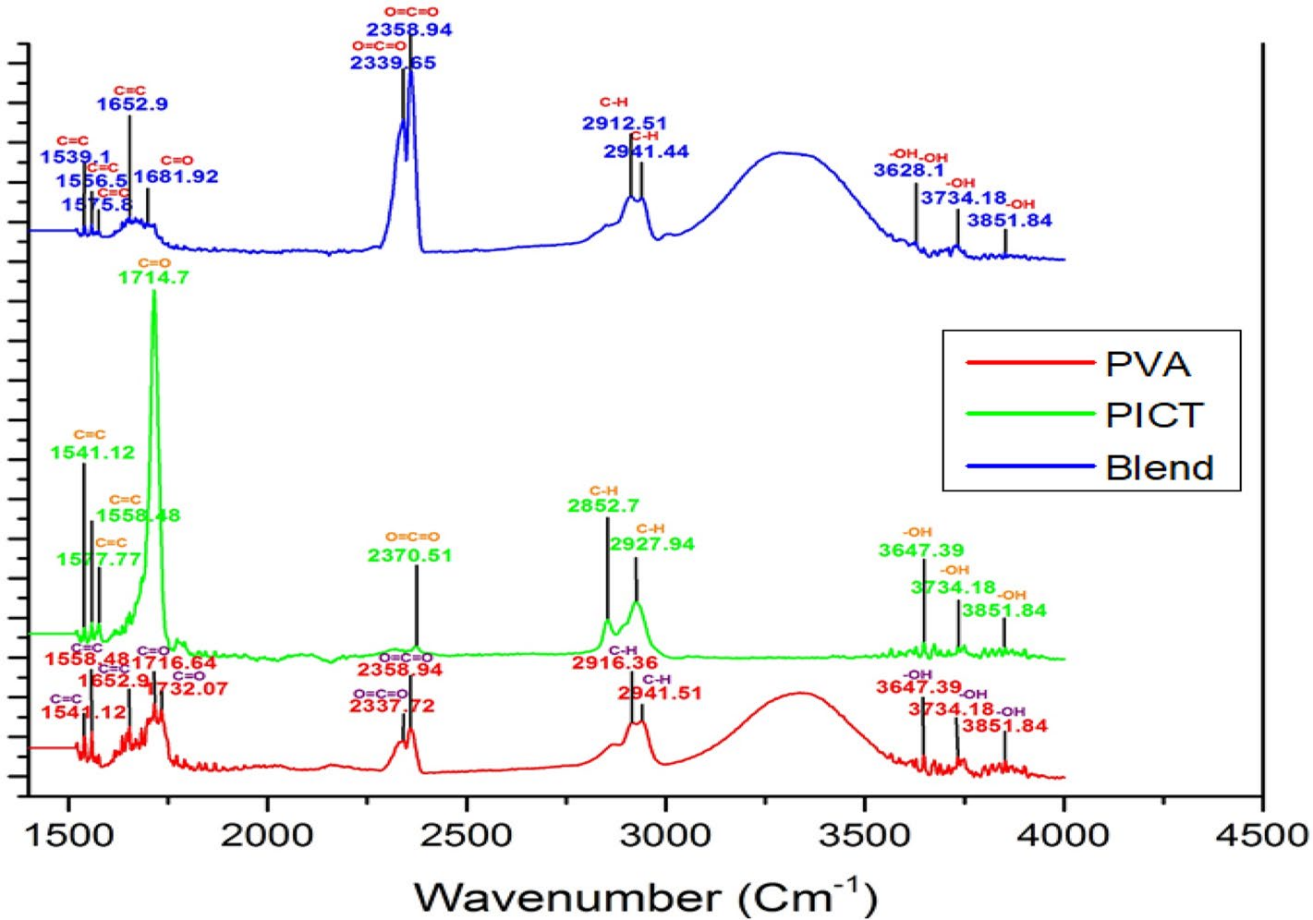
FT-IR spectra of PVA, PICT and PVA/PICT nanofibers.

Figure 5 shows the FTIR spectra of neat PVA nanofibers, neat PCIT nanofibers and blend (PVA: PCIT) nanofibers in the range from 1500 to 4000 cm^−1^. FTIR spectra of pure PVA and blended nanofibers are almost similar to each other. In the case of pure PVA and blended nanofibers, a broader band can be seen in the range of 3000 to 3500 cm^−1^ which indicates the presence of hydroxyl (–OH) group due to the hydrophilic nature of PVA polymer^1^. The peaks at 1541.12 cm^−1^, 1558.48 cm^−1^ (in case of PVA) while peaks at 1539.1 cm^−1^, 1556.5 cm^−1^, 1575.8 cm^−1^ (in case of the blend) indicates C=C bond stretching due to conjugated alkene. The peaks at 1652.9 cm^−1^ in both spectra (PVA and Blend) indicated C=C bond stretching due to cyclic alkene. The peaks at 1716.64 cm^−1^, 1732.07 cm^−1^ in the case of PVA indicate C=O bond stretching due to the presence of carboxylic acid and aldehydes group. The peak at 1681.92 cm^−1^ in the case of blend nanofibers indicates C=O bond stretching due to secondary amide linkage. The peaks at 2337.72 cm^−1^, 2358.94 cm^−1^ (in case of PVA) and the peaks at 2339.65 cm^−1^, 2358.94 cm^−1^ (in case of the blend) indicate O=C=O stretching due to the carbon dioxide. The peaks at 2916.36 cm^−1^, 2941.51 cm^−1^ (in case of PVA) and 2912.51 cm^−1^, 2941.44 cm^−1^ (in case of the blend) indicate the C-H bond stretching due to the alkane. The peaks at 3647.39 cm^−1^, 3734.18 cm^−1^, 3851.84 cm^−1^ (in case of PVA) and the peaks at 3628.1 cm^−1^, 3734.18 cm^−1^, 3851.84 cm^−1^ (in case of the blend) indicates O–H stretching due to the formation of alcohol. On the other hand, the spectra of PCIT nanofibers is quite different from that of the PVA and blended nanofibers. The highest and sharp peak in the case of PICT spectra can be seen at 1714.7 cm^−1^ which indicates C=O bond stretching due to carboxylic acid. The peaks at 1541.12 cm^−1^, 1558.48 cm^−1^, and 1577.77 cm^−1^ indicate the presence of C=C bond stretching due to conjugated alkene. It can be seen in the spectra that there is no peak of O–H is present between 3000 cm^−1^ to 3500 cm^−1^, like that of indicated in the case of PVA and blend nanofibers, this is due to the hydrophobic nature of PICT polymer as it contains a cyclic structure which is very stable against the moisture. PCIT spectra the peak at 1714.7 cm^−1^, 2370.51 cm^−1^, indicated C=O and O=C=O stretching respectively. Small peaks of –OH stretching appears in all three spectra indicated the presence of an alcoholic group^24^.

### Study of hydrophilic/hydrophobic behavior

**Figure 6:**
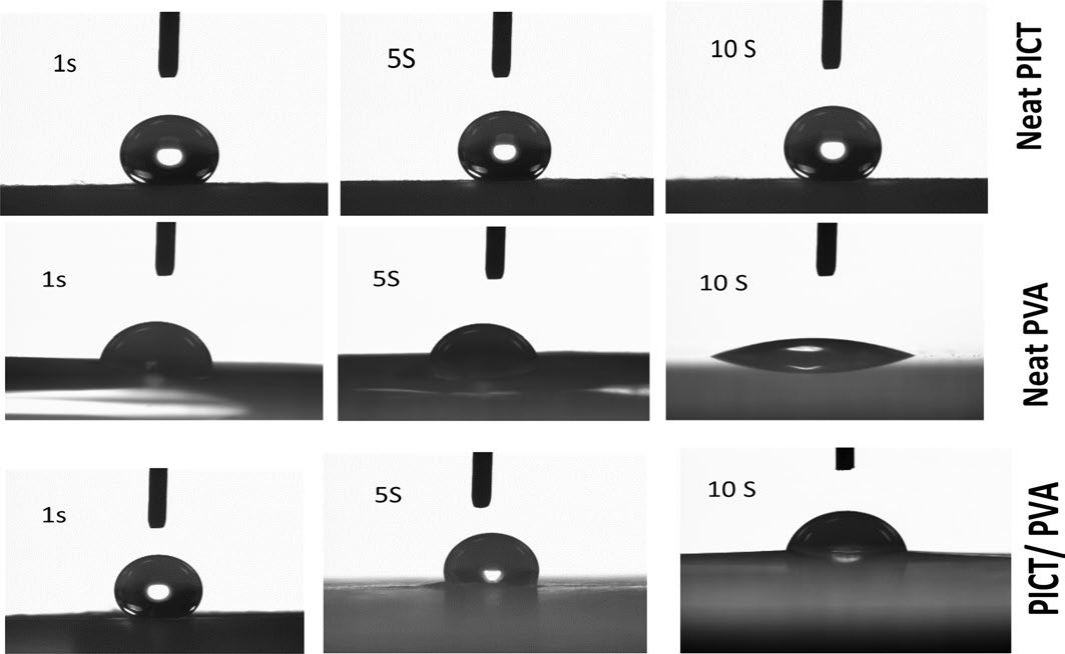
Study of water contact angle of PVA, PICT & PICT/PVA nanofibers.

**Figure 7:**
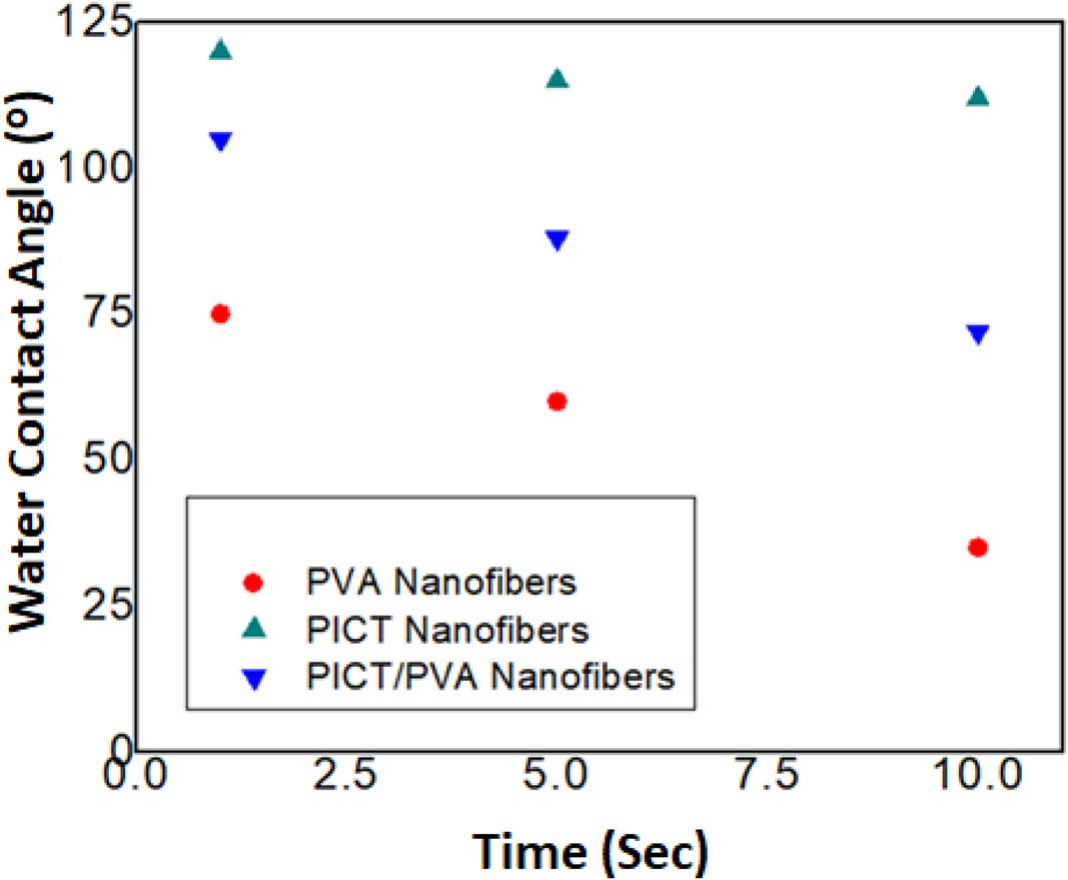
Study of water contact angle of PVA, PICT & PICT/PVA nanofibers.

To investigate the hydrophilic/hydrophobic behavior of neat PVA nanofibers, PVA/PICT nanofibers and PICT nanofibers, a state water contact angle test was performed as shown in Figs. 6, 7. It was analyzed that neat PVA has an appreciable water absorbency level which was 40°, 63° and 75° in 1 s, 5 s and 10 s, respectively. It shows that PVA has good hydrophilic behavior which is appropriate for the hydrophilic scaffold. On the other side, neat PICT nanofibers were analyzed and it was observed that PICT nanofibers have hydrophobic behavior because water contact angle was 123°, 118° and 111° in 1 s, 5 s and 10 s, respectively. But PVA/PICT nanofibers showed the water contact angle 105°, 88° and 72° in 1 s, 5 s and 10 s respectively. It means PICT/PVA nanofibers have the appropriate level of water absorbency as required for an ideal scaffold. The scaffold which must use in wound dressings, drug delivery, filtration of blood or urine must have an absorbency level from 70 to 100 water contact angle^25^. So proposed PVA/PICT nanofibers have the ideal level of absorbency as per requirements of scaffolds.

### Stress and strain behavior

**Figure 8:**
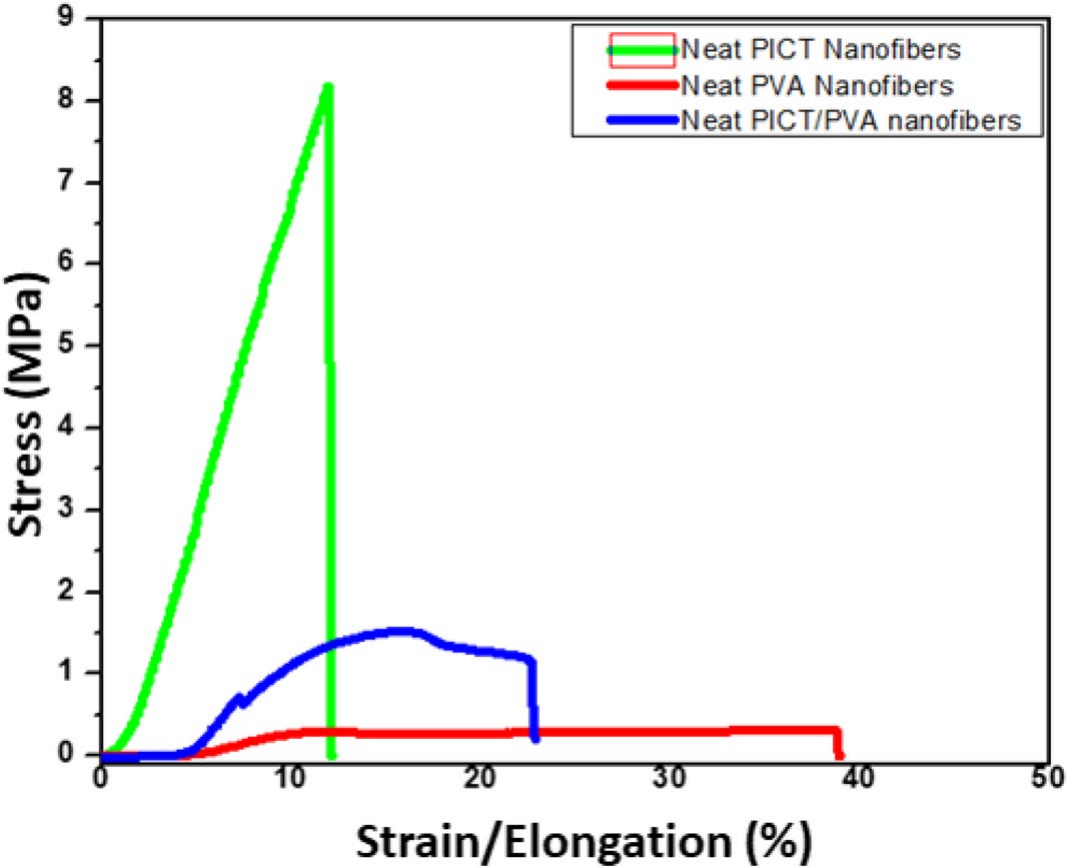
Stress and strain behavior of PVA, PICT & PICT/PVA nanofibers.

To investigate the stress and strain behavior of resultant nanofibers, samples were evaluated by a universal testing machine as shown in Fig. 8. It was confirmed that PVA has the highest elongation than PICT & PVA/PICT nanofibers but PVA/PICT has appreciable elongation and suitable stress and strain behavior. PICT nanofibers have the maximum ability to bear the stress up to 8.2 MPa but very poor behavior for elongation, after blending with PVA it showed the appreciable elongation up to 24% which 2 times of neat PICT. As discussed in the Fig. 2 that resultant scaffold was prepared by the co-electrospinning in which two types of nanofibers were entangled to each other by mechanical interlacement. Both types of nanofibers have different properties of stress strain behavior, but their blend showed the different behavior from them due to the interlacement of high elongated PVA nanofibers and high stiffed PICT nanofibers^24,25^. The behavior of PICT/PVA nanofibers is appropriate for the scaffold application because there is need of appropriate elongation and stiffness which was obtained by co-electrospinning of PVA and PICT polymers^26^.

### In-vitro assessment of scaffold

**Figure 9:**
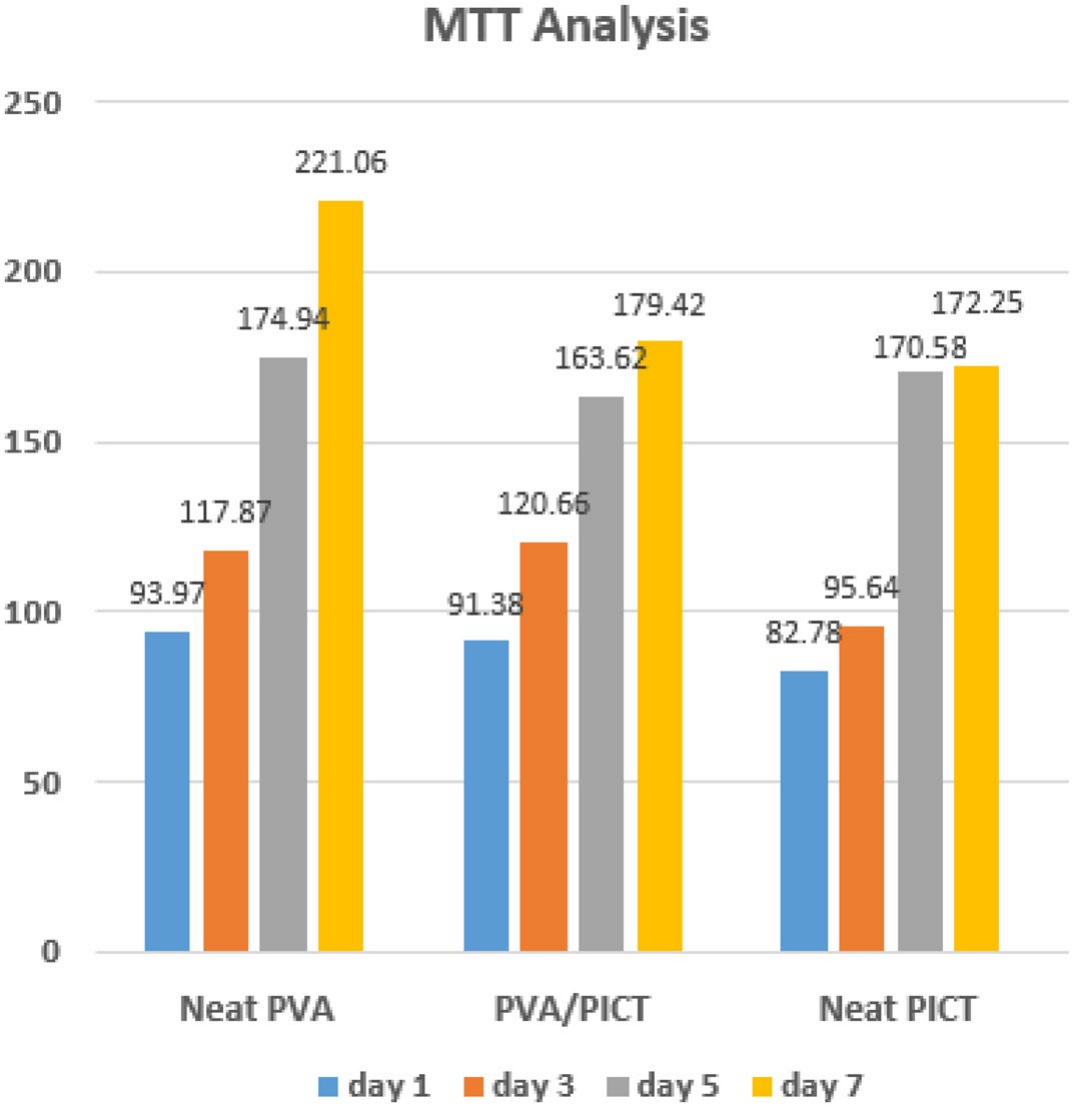
In-Vitro assessment of PVA, PICT & PICT/PVA nanofibers up to 7 days.

To investigate the toxicity of PVA nanofibers, PICT nanofibers and PICT/PVA nanofibers, MTT analysis was done as shown in Fig. 9. MTT study was done up to 7 days in line L929 with a two-time repeat in triplicate for each sample and cell line. After sterilization, the samples were cultured up to 7 days in DMEM/F12 with 10% FBS for L929. It was confirmed that all resultant samples don’t have toxicity but appreciable cell growth. Cell viability of all nanofibers was gradually increased day by day. Cell viability rate of PICT/PVA nanofiber scaffold was appreciable and was higher than both up to 3 days but after 7 days it was higher than neat PICT nanofibers and lower than neat PVA nanofibers as shown in Fig. 9. MTT analysis confirmed that through co-electrospinning, the resultant scaffold has the appreciable potential for use in tissue engineering due to its good cell culture and hydrophilic behavior^26^.

## Conclusion

Herein, successfully innovation of scaffold by co-electrospinning of super hydrophilic and superhydrophobic polymers was done. The resultant PICT/PVA nanofibers scaffold revealed the appreciable results of in-vitro, morphology, chemical interactions, wetting behavior and stress and strain behavior which were evaluated by concerned characterizations. PVA/PICT scaffold showed the cell growth and it was gradually increased as no day increased and the growth rate of cells in PVA/PICT nanofibers was higher than others up to 3 days but lower than neat PVA nanofibers up to 7 days but was higher than neat PICT nanofibers. Similarly, wetting behavior was appreciable as compare to PVA (very high, not good for various scaffold) and PICT nanofibers (very low, not good for various scaffold). Hence, it was concluded based on characterization results that PICT/PVA nanofibers can be used scaffold for tissue engineering to fulfill the stated and implied needs of the investors.
